# Dissolution and Precipitation Behaviour during Continuous Heating of Al–Mg–Si Alloys in a Wide Range of Heating Rates

**DOI:** 10.3390/ma8052830

**Published:** 2015-05-22

**Authors:** Julia Osten, Benjamin Milkereit, Christoph Schick, Olaf Kessler

**Affiliations:** 1 Chair of Materials Science, Faculty of Mechanical Engineering and Marine Technology, University of Rostock, Albert-Einstein-Str. 2, 18059 Rostock, Germany; E-Mails: benjamin.milkereit@uni-rostock.de (B.M.); olaf.kessler@uni-rostock.de (O.K.); 2 Polymer Physics Group, Institute of Physics, University of Rostock, Wismarsche Str. 43-45, 18057 Rostock, Germany; E-Mail: christoph.schick@uni-rostock.de

**Keywords:** continuous heating, differential scanning calorimetry (DSC), aluminium alloys, Al–Mg–Si, sheet, dissolution, precipitation, enthalpy change

## Abstract

In the present study, the dissolution and precipitation behaviour of four different aluminium alloys (EN AW-6005A, EN AW-6082, EN AW-6016, and EN AW-6181) in four different initial heat treatment conditions (T4, T6, overaged, and soft annealed) was investigated during heating in a wide dynamic range. Differential scanning calorimetry (DSC) was used to record heating curves between 20 and 600 °C. Heating rates were studied from 0.01 K/s to 5 K/s. We paid particular attention to control baseline stability, generating flat baselines and allowing accurate quantitative evaluation of the resulting DSC curves. As the heating rate increases, the individual dissolution and precipitation reactions shift to higher temperatures. The reactions during heating are significantly superimposed and partially run simultaneously. In addition, precipitation and dissolution reactions are increasingly suppressed as the heating rate increases, whereby exothermic precipitation reactions are suppressed earlier than endothermic dissolution reactions. Integrating the heating curves allowed the enthalpy levels of the different initial microstructural conditions to be quantified. Referring to time–temperature–austenitisation diagrams for steels, continuous heating dissolution diagrams for aluminium alloys were constructed to summarise the results in graphical form. These diagrams may support process optimisation in heat treatment shops.

## 1. Introduction

Al–Mg–Si alloys can be strengthened through precipitation hardening. The process consists of solution annealing, quenching, and ageing; thus, it contains heating, cooling, and isothermal steps, and thereby precipitation and dissolution reactions occur. The knowledge of the precipitation and dissolution behaviour of aluminium alloys is important for optimising heat treatment steps and acquiring information for simulation in the production chain. The necessity of investigating the heating behaviour of aluminium alloys shall be demonstrated with a few examples: aluminium sheet materials are coiled and solution annealing is performed in continuous annealing furnaces with very short solution annealing times (a few minutes, which allows production to be kept fast and cost-efficient). Knowledge of the dissolution behaviour over a wide dynamic range would help to select an appropriate heating rate, generate a full solution already during heating, and hence exhaust the full age-hardening potential. Another important field of application of dissolution is heat treatments which lead to an increase of plastic formability. This holds for forming processes such as tailored heat treated blanks (e.g., [1]), but also for joining processes such as laser-assisted clinching [2].

In recent years, the precipitation and dissolution behaviour during the ageing of Al–Mg–Si alloys has frequently been investigated through differential scanning calorimetry (DSC), occasionally in conjunction with microstructure analysis. One outcome of this has been that the highly complex precipitation sequence of Al–Mg–Si alloys is basically known (e.g., [3,4,5]). A simplified precipitation sequence from a saturated solid solution (sss) for Al–Mg–Si alloys could be described as: sss → cluster → GP zones → β′′ → β′ → β (Mg_2_Si). Nevertheless, the exact sequence significantly depends on the initial microstructural condition and heat treatment respectively as well as the alloy composition (*cf.* [6,7,8,9]). It is generally accepted that an increasing heating rate shifts the reactions towards higher temperatures (e.g., [10]). This phenomenon is often used to evaluate kinetic parameters such as the activation energy for specific precipitation reactions (e.g., [11,12,13]). A number of different methods exist to evaluate such kinetic parameters [14,15]. All of these methods are based on the assumption that the same amount of alloying elements is transferred for varying heating rates. However, the variation of the heating rate in these methods has been limited to a relatively narrow dynamic range so far. The availability of DSC curves is limited to a typical heating rate range around 10 K/min (about 0.2 K/s). In technological applications, the heating rate will vary significantly depending on heating technique and part dimensions. Therefore, it is indispensable to investigate a multiplicity of heating rates in a wide dynamic range. Moreover, DSC data in previous studies are hard to compare because the evaluation of those curves is not consistent. Heat flow was illustrated frequently instead of excess specific heat capacity, which is normalised for scanning rate and sample mass and therefore more comparable.

The purpose of this work is to investigate the sequence of the precipitation and dissolution of Al–Mg–Si alloys in a wide dynamic range during heating by means of highly precise *in-situ* DSC analysis to make this information available for heat treatment shops as well as for heat treatment simulation.

## 2. Materials and Methods

### 2.1. Analysed Aluminium Alloys and Investigated Initial Conditions

For the experimental procedure, four wrought alloys of the alloy system Al–Mg–Si were chosen. EN AW-6005A and EN AW-6082 were supplied as extruded profiles, EN AW-6016 and EN AW-6181 instead as sheets. The chemical compositions—analysed by optical emission spectrometry (OES)—are given in Table 1. The alloy contents comply with the standard DIN EN 573-3, except aluminium alloy 6181, which has a slightly higher copper content. In addition, 99.9995% pure aluminium was used as reference material.

**Table 1:** Mass fraction of alloying elements of the investigated materials in %.

Aluminium Alloy	Mass fraction in %							
	Si	Fe	Cu	Mn	Mg	Cr	Zn	Ti
EN AW-6005A	0.67	0.23	0.03	0.41	0.59	0.01	0.02	0.02
DIN EN 573-3	0.5–0.9	≤0.35	≤0.3	≤0.5	0.4–0.7	≤0.3	≤0.2	≤0.1
EN AW-6082	0.73	0.22	0.05	0.48	0.61	0.003	0.01	0.02
DIN EN 573-3	0.7–1.3	≤0.5	≤0.1	0.4–1.0	0.6–1.2	≤0.25	≤0.2	≤0.1
EN AW-6016	1.15	0.24	0.07	0.07	0.40	0.022	0.007	0.04
DIN EN 573-3	1.0–1.5	≤0.5	≤0.2	≤0.2	0.25–0.6	≤0.1	≤0.2	≤0.15
EN AW-6181	0.85	0.33	0.18	0.06	0.77	0.009	0.021	0.01
DIN EN 573-3	0.8–1.2	≤0.45	≤0.1	≤0.15	0.6–1.0	≤0.1	≤0.2	≤0.1

The dissolution and precipitation behaviour strongly depends on the initial microstructural state or heat treatment state. Therefore, different heat treatment states were adjusted, naturally aged (T4), artificially aged (T6), overaged (OA), and soft annealed (SA). The corresponding time–temperature profiles are listed in Table 2. The DSC measurements of the alloys EN AW-6016 T4 and EN AW-6181 T4 were done at the same state of 7 days ageing time, since the samples were frozen at –80 °C after natural ageing prior the DSC measurements.

**Table 2:** Heat treatment parameters of the investigated material.

Heat treatment	Step I	Step II	Step III
T4	Solution annealing 540 °C 20 min.	Water quenching	Natural ageing 25 °C 7 days ^*^
T6			Artificial ageing 180 °C 4 h
Overaged (OA)			Artificial ageing 200 °C 10 h
Soft annealed (SA)		98 h furnace cooling	-

^*^ EN AW-6005A: 6–8 days; EN AW-6082: 2 months.

### 2.2. Thermal Analysis through DSC

The heating experiments were carried out in two different devices adapted to different heating rate ranges. For rates between 0.01 and 0.1 K/s a heat-flux DSC (Setaram DSC 121, SETARAM Instrumentation, Frankfurt am Main, Germany) and for rates between 0.3 K/s to 5 K/s a power-compensated DSC (PerkinElmer Pyris 1 DSC, PerkinElmer, Hamburg, Germany) was used. The samples have a cylindrical geometry, with dimensions of Ø6.1 mm × 21.65 mm used for measurements in the Setaram DSC 121 and of Ø6.4 mm × 1.0 mm for measurements with the Perkin-Elmer Pyris 1 DSC. Ensuring the symmetry of the whole equipment’s arrangement during the measurements is the determining factor for the quality of DSC curves. Therefore, not reacting reference samples of pure Al with the same geometries were used.

In order to obtain precisely evaluable DSC curves, as a first step the device-specific heat-flow curvature must be eliminated from the raw data. Thus, a baseline measurement is subtracted from the sample measurement. Not only does the symmetry within one measure have to be preserved, but also the symmetry of successive sample and baseline measurements. Therefore, during the sample measurement the alloyed sample is scanned against a not reacting reference sample, while the baseline measurement is made by scanning a pure Al sample against a pure Al sample. To ensure that this procedure is appropriate, it is necessary to have steady-state conditions at the beginning and at the end of the measurements. Due to thermal inertia of the devices, it is useful to start and end the measurement with an isothermal step (this is also required for the heat capacity calculation with the device software). These isothermal steps serve the equilibration of the temperature of the sample. The starting isotherm could be described as a *low temperature isotherm*, in contrast to the ending isotherm, which is a *high temperature isotherm*. In particular, the quality with which the heat-flow values are matched between the high temperature isotherms of the baseline and the sample is a quality characteristic of a DSC measurement. The values of the heat-flow differences between the sample- and the baseline measurement are device dependent. With heat-flux DSCs such as the Calvet-type Setaram DSC 121, significantly better results are generally obtained with respect to this issue.

To avoid baseline drift problems sample measurements and baseline measurements were recorded back-to-back. For slow heating rates (0.01–0.1 K/s) three sample and one baseline measurement were arranged. In contrast, six sample and three baseline measures were made for fast heating (0.3–5 K/s) rates. For measurements carried out in the PerkinElmer Pyris 1 DSC, pure nitrogen was used as a purge gas to ensure efficient heat transfer for improved sensitivity. Device-specific, no purge gas was used in the Setaram DSC 121, *i.e.*, heating was carried out in dry air.

We aim to perform a quantitative evaluation of the DSC curves. Therefore, we have to ensure that the zero level of the evaluated curve (frequently called the baseline, which should not be confused with the baseline measurement) is absolutely straight and possesses a value that is equal to zero. This is not necessarily the case, even if the measurement is performed with the best possible accuracy. Several circumstances can lead to a remaining zero-level curvature. For example, varying positions/rotations of the furnace lids or slight variations of the positions of the samples inside the furnaces can cause curve-bending effects (see e.g., [16]). In addition, the sample might have a slightly different surface colour compared with the reference sample. This will lead to a different uptake of heat radiation, which influences the heat flow particularly in heat-flux DSCs at high temperatures (see e.g., [17]). This metrological issue is reduced by covering the samples with standard aluminium crucibles, as this minimises the influence of different heat radiation behaviours of different sample surface colours on the heat flow signal and allows the reception of a good accordance of the curves of the sample measurement and the baseline [17]. Nevertheless, slight remaining bending will remain in several cases. This must be eliminated to allow accurate qualitative and quantitative evaluations. Primarily, and only if the zero level is known to within a good approximation, one can qualitatively distinguish between endo- and exothermal reactions. Moreover, quantitative evaluation, for instance of the enthalpy transformed by the integration of the excess specific heat capacity curves, is only appropriate if the zero-level baseline is truly zero. An appropriate procedure to ensure this zero-level baseline quality will be explained below.

### 2.3. Specifics for Using Modified Sample Geometries during DSC Measurements

**Figure 1:**
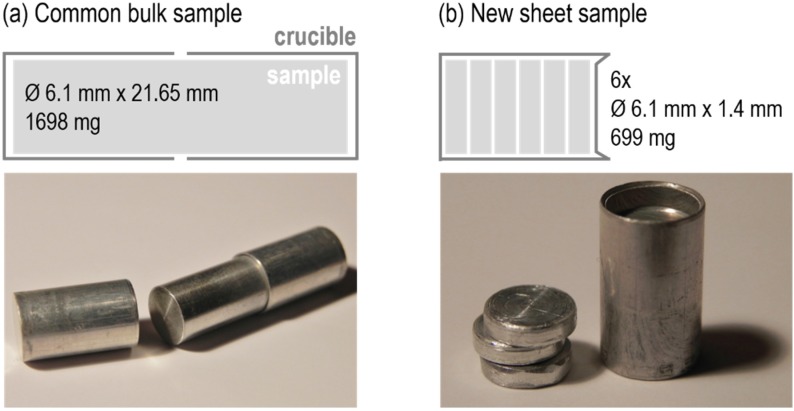
Schematics for the used common (**a**) and new sheet (**b**) sample with dimensions and masses as well as pictures of EN AW-6005A samples with crucibles.

In this work a challenge was using sheet materials of EN AW-6016 (sheet thickness t = 1.25 mm) and EN AW-6181 (t = 1.5 mm) for measurements in the Setaram DSC 121. To get appropriate sample masses, several thin sheet discs needed to be stacked to arrive at the final samples. A stacked sample might be a challenge due to the significantly increased surface-to-volume ratio of the sample. In particular, this might lead to increased baseline bending as a result of heat-radiation effects. Preliminary investigations with EN AW-6005A exhibited the possibility of using common bulk samples on the one hand and stacked thin sheet samples on the other hand. Figure 1 shows a schematic and a picture of the two different sample dimensions used. Characteristics of the samples are different geometries and masses. While a common bulk sample is covered by two standard aluminium crucibles and weighs about 1,698 mg, the new stacked sheet sample fills only one crucible, weighs about 700 mg, and is capped with an aluminium lid. The reference samples were prepared in the same way to retain the symmetry of the measurements.

**Figure 2:**
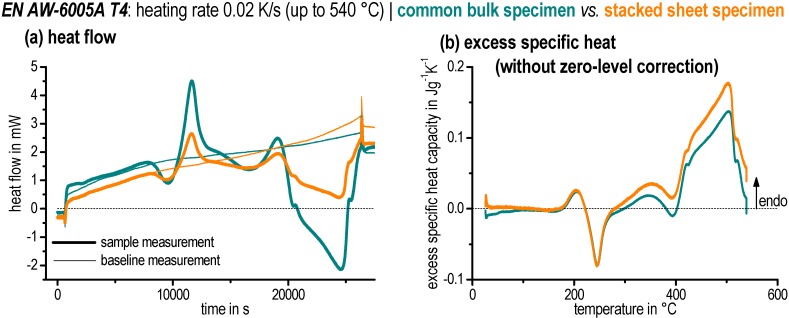
Comparison of different specimen types during heating of EN AW-6005A in a Setaram DSC 121: (**a**) heat-flow curves; and (**b**) excess specific heat capacity curves (normalised heating curves).

The recorded heat flow curves of both sample measurements and baseline measurements as well as the normalised excess specific heat capacity curves can be seen in Figure 2. A constant heating rate of 0.02 K/s up to 540 °C was used in this example. Excess specific heat capacity curves allow a comparison of the heating curves with respect to different heating rates and mass. The curves match very well, *i.e.*, the stacked discs method has been developed successfully for DSC measurements on sheet metal.

### 2.4. Evaluation of Heat Flow Curves

First, as expected, it is noticeable in Figure 2a that the measured heat flows of the stacked sample are significantly lower due to the reduction in sample mass. The difference of the high temperature isotherms for the common bulk specimen is approximately 0.2 mW. In contrast, the difference for the new stacked specimen is in the region of about 0.6 mW. Although both results are quite satisfactory it can be seen that the curve values after baseline-subtraction are not necessarily zero, even if the reactions seem to be finished in the sample. In addition, the excess specific heat capacity (Figure 2b) displays a good qualitative accordance for both specimens. However, it becomes apparent that the remaining curvature of the corresponding zero levels is different.

This remaining zero-level curvature potentially can be eliminated by subtracting a polynomial (zero-level polynomial). However, for this the start and end of the measured curves should exhibit sections where no heat was exchanged by microstructural changes within the sample—*i.e.*, reaction-free zones are required to allow the fitting of a zero-level polynomial. This requirement is not fulfilled in the example in Figure 2b. Nevertheless, this problem could be overcome by heating to higher temperatures —in particular some 10 K above the solvus temperature of the equilibrium phases—which would consequently lead to curve progression with significant and expanded reaction-free sections.

**Figure 3:**
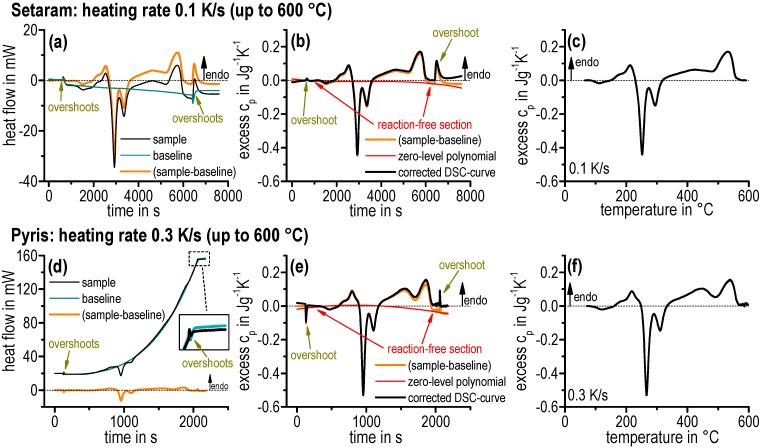
Data handling to obtain an undistorted normalised DSC heating curve by means of data from several devices using the example of EN AW-6181 T4. (**a**–**c**) Setaram DSC 121. (**d**–**f**) PerkinElmer Pyris 1 DSC.

The data evaluation procedure is shown in Figure 3 with raw data from the two DSC devices measuring EN AW-6181 T4. In order to indicate the comparability of both DSC types, similar heating rates of 0.1 K/s (Figure 3a–c) and 0.3 K/s (Figure 3d–f) were chosen. The consideration of the excess specific heat capacity provides significant advantages for the characterisation of the slight dissolution and precipitation reactions in contrast to heat flow. Evaluating the excess specific heat capacity, sample heat flow (${\dot{Q}}_{\text{sample}}$) and baseline heat flow (${\dot{Q}}_{\text{baseline}}$) have to be subtracted (Figure 3a,d). The remaining zero-level curvature was corrected by subtracting a third degree polynomial (see Figure 3b,e) after normalising through sample mass (*m*) and scan rate (β). This method of calculating the excess specific heat capacity is generally applied to compare different heating rates and different sample masses [18]:

(1) $$c_{p}=\frac{{\dot{Q}}_{\text{sample}}-\;{\dot{Q}}_{\text{baseline}}}{m∙\text{β}}$$

The zero-level polynomial is fitted to the reaction-free zones. The curve correction should be performed on curves in the same scale. Moreover, the correction should always start on curves with the largest measuring effects due to proper estimation of the curvature. However, this procedure should only be used if it is certain that reactions and reaction-free sections can be distinguished from each other, e.g., by comparing other curves of slightly different heating rates. Nevertheless, this method is a subjective procedure and must be handled with care.

Another important fact during evaluation is shown in Figure 3a,b and 3d,e. Changing the heating rate significantly, e.g., from a constant heating rate to the isothermal soaking at high temperatures, unavoidably leads to heat-flow artefacts. A so-called overshoot peak can be seen depending on the DSC device. These artefacts must be excluded from evaluation.

Subsequently, the resulting de-bended curve can be displayed as a function of temperature (Figure 3c,f). In the course of obtaining the resulting DSC heating curves, the endothermic reactions by definition correspond to the positive number range while the exothermic reactions correspond to the negative number range [18]. The differences between the start isothermal and end isothermal for the PerkinElmer Pyris 1 DSC (Figure 3d) have a greater magnitude than for the Setaram DSC 121 due to the device. Nevertheless, both evaluated excess heat capacity curves ultimately show a similar behaviour, indicating a good comparability of the two DSC devices used.

**Figure 4:**
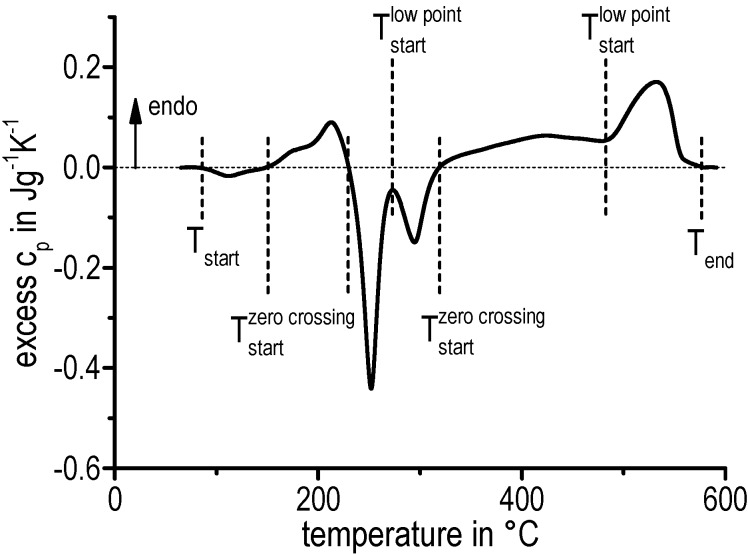
Separation of the individual peaks and defining onset and endset temperatures by using the example of EN AW-6181 T4 heated with 0.1 K/s employing a Setaram DSC 121.

The generation of continuous heating dissolution diagrams is based on the interpretation of the heating curves (excess cp as a function of temperature). Due to the overlapping of the dissolution and precipitation reactions, a first approach has been used to determine onset and endset temperatures. The onset and endset temperatures of each precipitation and dissolution reaction respectively is defined by the zero crossing (see Figure 4). The start temperature $T_{start}$ of the first reaction at low temperatures as well as the end temperature $T_{end}$ of the last reaction at the highest temperature could be interpreted as the true start/end temperatures. The temperatures of the individual peaks $T_{start}^{zero\;crossing}$ are simplifications, as overlapping of the reactions is not considered. The superimposed peaks without zero crossing are a special case of evaluating onset temperatures. These temperatures $T_{start}^{low\;point}$ are defined by the minimum value between both peaks. The investigated heating curves as well as the onset and endset temperatures of the different dissolution and precipitation reactions are finally plotted in a temperature–time diagram.

The curve area integration over the whole temperature range provides another quantitative evaluation method for heating curves. The complete enthalpy change distinguishes the amount of existing precipitations in the initial condition just before the heating scan.

## 3. Results and Discussion

### 3.1. Excess Specific Heat Capacity Curves

**Figure 5:**
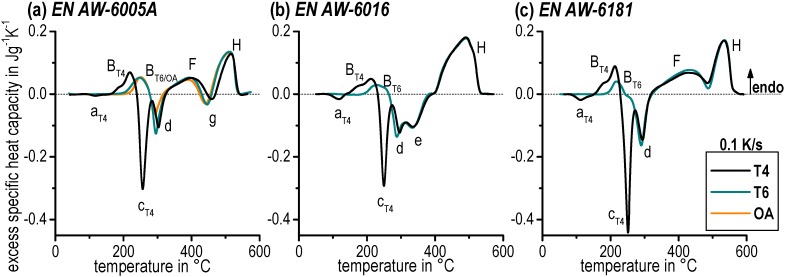
Continuous heating DSC curves at 0.1 K/s for various initial conditions (**a**) EN AW-6005A – T4, T6 and OA. (**b**) EN AW-6016 T4 and T6. (**c**) EN AW-6181 T4 and T6.

In general, energy has to be supplied if chemical bonds dissociate, which refers to an endothermic reaction. However, exothermic reactions display energy release and indicate that new chemical bonds have been formed. Aluminium alloy dissolutions correspond to endothermic reactions while precipitations belong to exothermic reactions. Endothermic dissolutions are shown by deviations exceeding the zero level, which is displayed as a dashed straight line (see Figure 5). Exothermic precipitations are represented by deviations below the zero level. The interpretation of those measurements might be very difficult. This holds particularly for heating experiments on age-hardened aluminium alloys due to a sequence of alternating endothermic dissolution and precipitation exothermic reactions. In addition, superposition of different reactions can emerge. As will be shown below, this involves the danger of significant misinterpretation during the quantitative interpretation of such DSC curves.

Figure 5 shows the heating DSC curves with an exemplary rate of 0.1 K/s for the alloys EN AW-6005A, EN AW-6016, and EN AW-6181 comparing the different initial conditions (T4, T6, and OA). The curves for EN AW-6082 are very similar to EN AW-6005A. The results demonstrate the complexity of the alternating endothermic and exothermic reactions caused by the precipitation sequence [3,4,5]. Ohmori *et al.* [10,19,20] as well as Birol [21,22] examined the heating behaviour of Al–Mg–Si alloys through DSC and showed a similar alternation of exothermic and endothermic peaks.

The excess specific heat capacity curves of all three alloys for the initial condition (T4) start with an exothermic peak *a_T4_* caused by the remaining potential for the formation of clusters, which were not formed during natural ageing before. For a higher content of Mg and Si, two superimposed cluster reactions occur [23]. This could be the reason for the intense peak *a_T4_* for the alloys EN AW-6016 T4 and EN AW-6181 T4. The considerably higher content of Si in those alloys causes an accelerated precipitation reaction at lower temperatures in comparison to EN AW-6005A. In addition, it should be noted that there is no exothermic reaction *a_T4_* for the alloy EN AW-6082 T4 due to the fact that natural ageing lasted about 2 months, resulting in no remaining potential for cluster formation.

Endothermic reaction *B_T4_* appears next and is generally interpreted as the dissolution of clusters [10] and, with respect to natural ageing, as the dissolution of GP zones [24]. The two-step shoulder in peak *B_T4_* indicates such overlapping dissolution reactions.

The following exothermic peaks *c_T4_* and *d* are frequently interpreted as precipitation of β′′ and β′ phases [5,25], respectively, for the T4 condition. A special case is the exothermic peak *e* of the alloy EN AW-6016 T4. This likely corresponds to the precipitation of B′, which is established in combination with β′ as well as being related to a high Si:Mg ratio [25]. The Si:Mg ratio of the alloy EN AW-6016 is more than twice as high as the other investigated aluminium alloys.

It is obvious that the following sequence of the peaks *F*, *g*, and *H* could not be clearly identified for every investigated alloy. The reasons are concurrent dissolution and precipitation reactions – a first hint of the serious problem of quantitative interpretation of such DSC curves. The peaks *F*, *g*, and *H* detected in EN AW-6005A correspond to the dissolution of the precursor phases such as β′′, β′ and B′. The subsequent occurring peak *e* should correspond to the precipitation of the equilibrium phase β (Mg_2_Si). The final peak *H* corresponds to the final dissolution of all remaining phases, predominantly β (Mg_2_Si). *F*, *g*, and *H* heat signals overlap significantly, so that for EN AW-6181 the exothermic effect of β (Mg_2_Si) precipitation only appears as a local minimum in a larger endothermic dissolution peak. Even more drastic for EN AW-6016 is the fact that the separation of those three reactions is not possible at all. Thus, for example, peak *g* could not be labelled for the alloys EN AW-6016 or EN AW-6181 (Figure 5b,c).

The T6 artificially aged conditions have a similar sequence of exothermic and endothermic reactions as the T4 conditions of each alloy. The specifics are non-existent peaks *a* and *c* as well as the modified peak *B*. This is due to the fact that GP zones and β′′ had been previously precipitated in the T6 condition. These particles are dissolved at peak *B_T6_*. The further sequence correlates with the development of the T4 natural aged condition.

The slightly modified curve of the OA condition of EN AW-6005A compared with the T6 condition shows a less intense reaction *d*. β′ particles were already precipitated at overaged condition, with the result that the potential for forming new precipitations while heating was lower.

It is also conceivable that precipitation transformations occur during heating, e.g., transformation of β′→β or transformation of pre-β′′ → β′′ might occur [25,26,27].

### 3.2. Dissolution and Precipitation Behaviour in a Wide Range of Heating Rates

**Figure 6:**
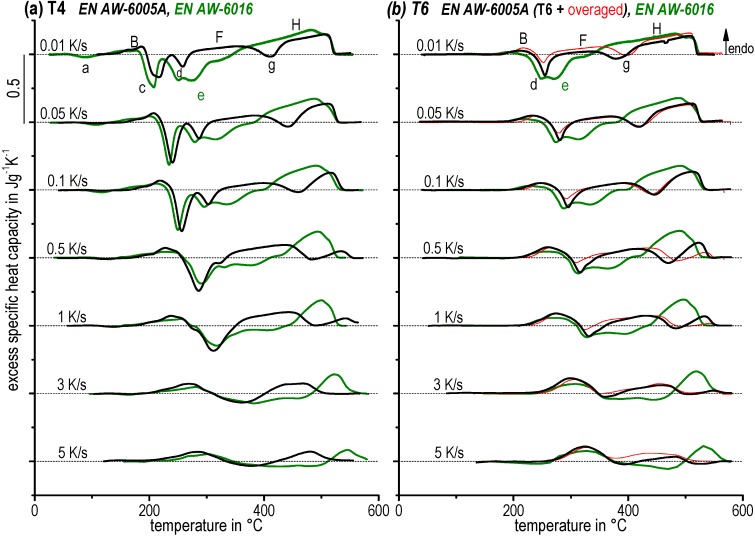
Selected heating curves of EN AW-6005A and EN AW-6016 for the initial conditions (**a**) T4. (**b**) T6 (and overaged).

Figure 6 shows selected excess specific heat capacity curves of the alloys EN AW-6005A and EN AW-6016 for the T4 and T6 initial conditions (and OA occasionally).

The DSC curves are arranged in descending order of increasing heating rate. In general, the peaks shift to higher temperatures as the heating rate increases. For example, the peak temperature of the precipitation reaction *c_T4_* of the alloy EN AW-6005A shifts about 160 K from 220 °C during heating at 0.01 K/s up to 380 °C during heating at 5 K/s. Further, the peak areas decrease as heating rate increases due to the suppression of diffusion processes. The precipitation of clusters (peak *a*) is suppressed completely at higher heating rates. Similar observations has been reported in Pogatscher *et al.* [28]. Higher heating rates lead to an incomplete precipitation sequence. The dissolution and precipitation reactions run earlier at slower heating rates for the alloy EN AW-6016, possibly due to its higher Si content as well as its higher Si:Mg ratio. This holds for both T4 and T6 initial conditions.

**Figure 7:**
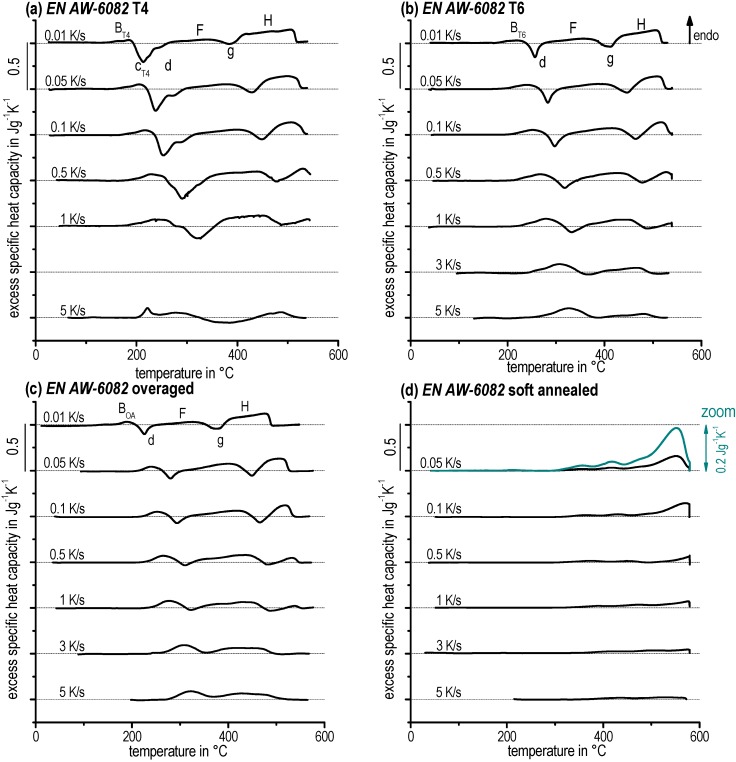
Continuous heating curves for EN AW-6082 with different initial conditions (**a**) T4. (**b**) T6. (**c**) Overaged. (**d**) Soft annealed.

**Figure 8:**
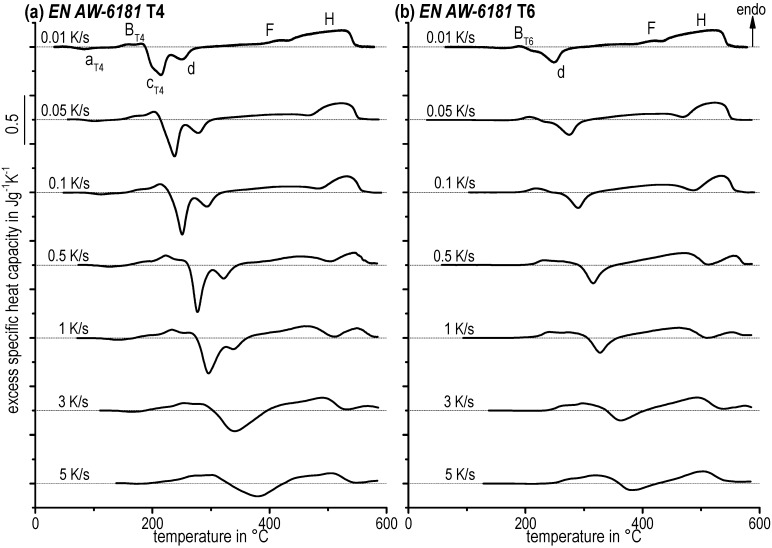
Continuous heating curves for EN AW-6181 with (**a**) T4 and (**b**) T6 initial condition.

Characteristic heating curves for the alloy EN AW-6082 (T4, T6, OA, SA) could be observed in Figure 7 as well as for EN AW-6181 (T4, T6) in Figure 8.

Both, precipitation and dissolution are diffusion-controlled reactions and therefore it is probable that they are increasingly suppressed as heating rate increases. This can be observed, e.g., following the final dissolution peak *H* over the entire heating rate range. Peak *H* is significantly suppressed as heating rate increases. Nevertheless, e.g., the precipitation peaks *c* and *d* of the T4 initial state seem to increase their peak area as heating rate increases. However, the underlying reactions must be suppressed. The seeming increase of the related peak areas can only be ascribed to changes in the degree of overlapping by endo- and exothermic reactions. It seems possible that the kinetic shifts in temperature are different. Hence, a different shift in temperature of superimposed dissolution and precipitation will lead to different peak ratios. Another possible explanation is that the kinetics of precipitation reactions is slower compared to the kinetics of dissolution reactions. This is because precipitation requires long-range diffusion as opposed to more localised dissolution. Thereby, precipitation reactions might be suppressed more strongly than dissolution reactions. The latter seem to be the case for all investigated conditions here, as at faster heating rates endothermic dissolution reactions clearly dominate the DSC curves.

Therefore, these overlapping and superposition issues imply that evaluation of kinetic parameters, e.g., for precipitation reactions based on DSC heating curves, must be handled with care. Some of the evaluation methods for kinetic parameters, such as the Kissinger method, utilise a single peak for evaluation—which, based on the above results, obviously brings along the danger of misinterpretation. Moreover, all methods for evaluating the kinetic data from DSC heating curves are based on the assumption that the transformed fraction of alloying elements is constantly independent of the varying heating rates [14,15,29]. The basic assumption also does not hold—in particular for a variation of heating rates in a wide dynamic range. This leads to the future task of developing new methods for the evaluation of kinetic parameters. That task might be solved by kinetic modelling. Some available models even allow the combination of both precipitation and dissolution to model the whole DSC heating curve from room temperature up to the solvus temperature (e.g., [30,31,32,33,34]).

**Figure 9:**
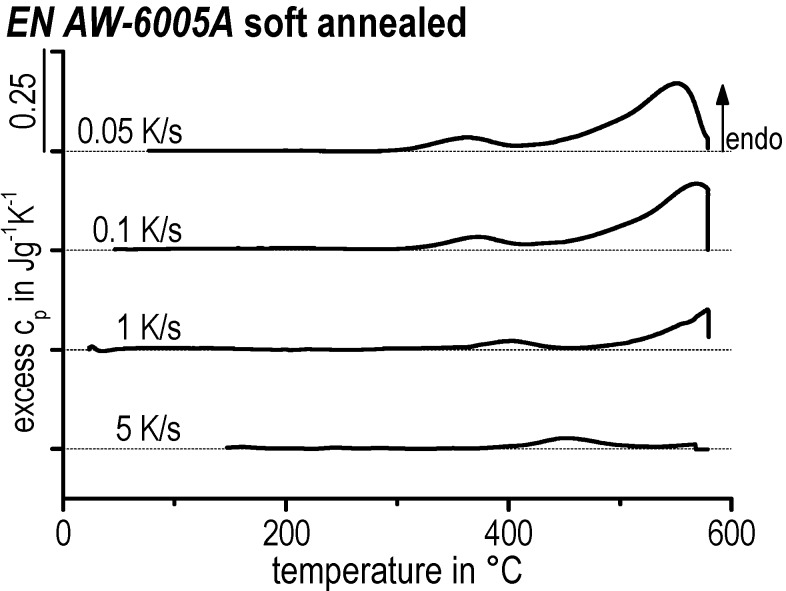
Selected heating curves of EN AW-6005A for the SA initial condition.

In Figure 9, selected heating curves for alloy EN AW-6005A in the SA initial condition are plotted. During heating after soft annealing, only dissolution reactions occur. During soft annealing, nearly all alloying elements will precipitate predominantly to incoherent β (Mg_2_Si) particles, but also to a minor fraction of semi-coherent β′ and/or B′ precipitates [35]. Such precipitations will dissolve during heating. For a heating rate of 0.05 K/s the dissolution is probably just completely finished at 580 °C—it can be seen in Figure 9 that the excess c_p_ beyond the final dissolution peak just drops back to zero. At least for the faster heating rates investigated, the dissolution reaction is incomplete. Thus, a critical heating rate applied at a certain temperature for complete dissolution exists. After faster heating to this particular temperature, additional soaking will be needed to solve all relevant alloying elements. Alloy EN AW-6082 was also investigated after soft annealing (see Figure 7d).

### 3.3. Continuous Heating Dissolution Diagrams

**Figure 10:**
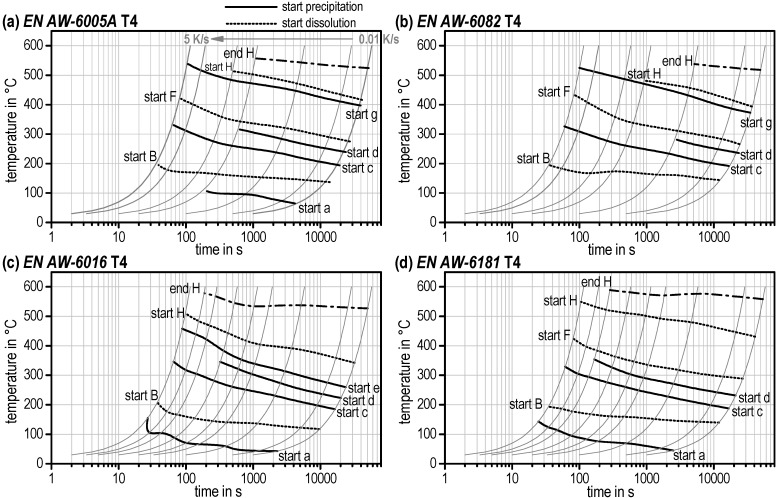
Continuous heating dissolution diagrams for the T4 initial condition of several aluminium alloys (**a**) EN AW-6005A. (**b**) EN AW-6082. (**c**) EN AW-6016. (**d**) EN AW-6181.

By entering the different start and end temperatures of the dissolution and precipitation reactions together with the varying heating curves in a temperature–time diagram, continuous heating dissolution diagrams for a wide range of heating rates could be created. Figure 10 displays the diagrams of all investigated Al–Mg–Si alloys for the T4 initial condition. These diagrams allow estimation of corresponding temperatures for dissolution or precipitation reactions during heating rates of 0.01 to 5 K/s and make these data available for heat treatment shops.

**Figure 11:**
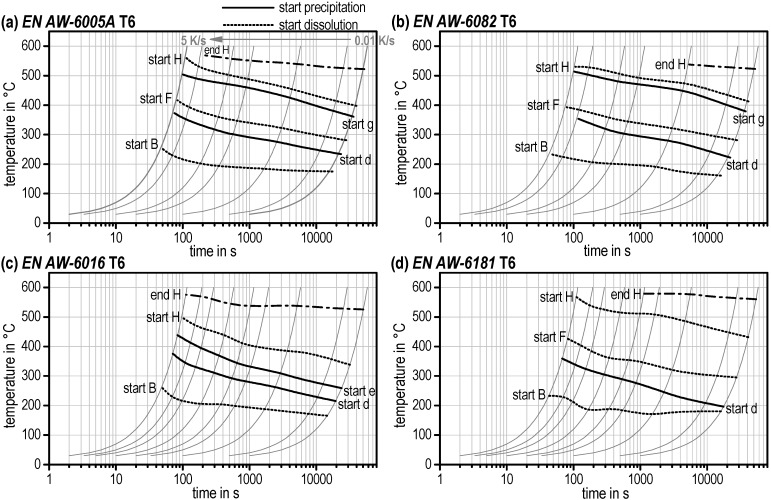
Continuous heating dissolution diagrams for the T6 initial condition of several aluminium alloys (**a**) EN AW-6005A. (**b**) EN AW-6082. (**c**) EN AW-6016. (**d**) EN AW-6181.

**Figure 12:**
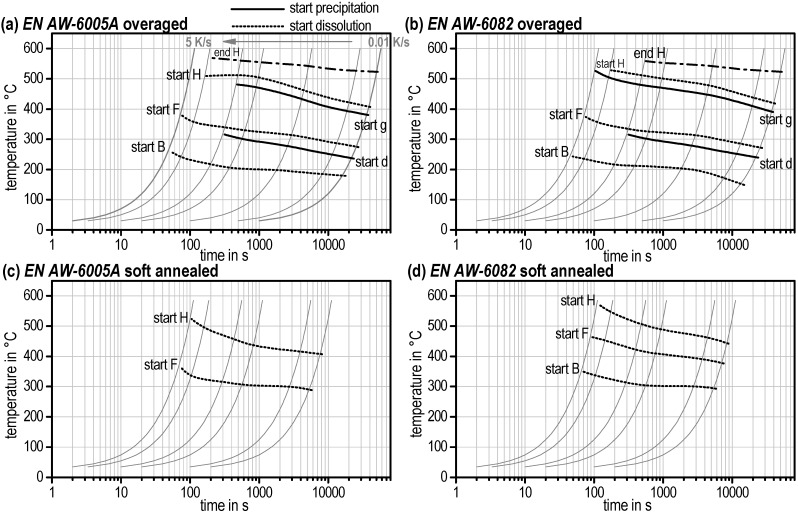
Continuous heating dissolution diagrams for (**a**) EN AW-6005A overaged; (**b**) EN AW-6082 overaged; (**c**) EN AW-6005A soft annealed; (**d**) EN AW-6082 soft annealed.

It should be mentioned again that these start temperatures were qualified by zero crossing (partial local minimum) of the DSC curves. The very first start temperature of reaction *a* and reaction *B* as well as the final end temperature of reaction *H* are physical true. The start temperatures of all other reactions are influenced by the evaluation method. The continuous heating dissolution diagrams for the T6 initial condition can be seen in Figure 11 as well as for OA and SA alloys EN AW-6005A and EN AW-6082 in Figure 12. 

One important detail of those diagrams regarding solution annealing shall be explicitly mentioned. Reaction *H* describes the final dissolution of all remaining phases in the aluminium solid solution. It is obvious that the temperatures “start *H*” and “end *H*” are not reached for all alloys, initial microstructures, and heating rates (e.g., no end *H* for EN AW-6082 for T4 and T6 above approx. 0.5 K/s). Here, dissolution has not been completed at the end of the heating step and will continue in the soaking step.

### 3.4. Enthalpy Change

**Figure 13:**
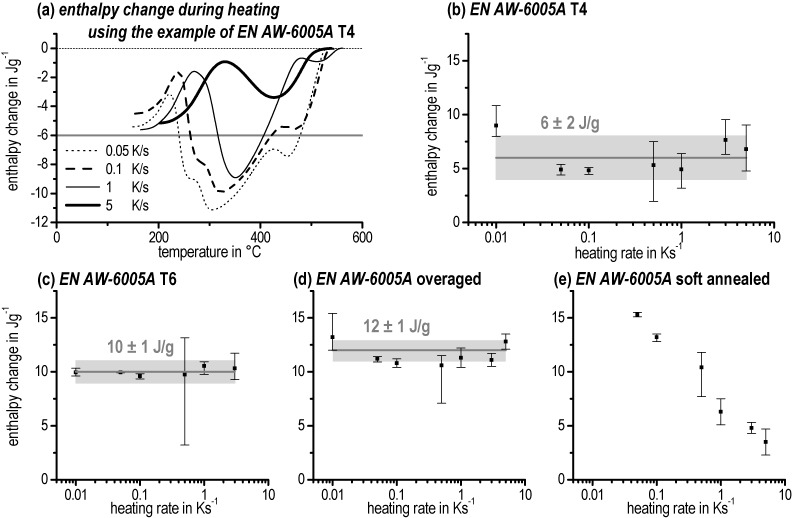
Enthalpy change of EN AW-6005A (**a**) T4, development of enthalpy change during heating with different heating rates. (**b**) T4. (**c**) T6. (**d**) Overaged. (**e**) Soft annealed.

The integral of the heating curves – covering the full temperature range – gives information about the heat consumed by the dissolution of the pre-existing initial microstructural state. The course of enthalpy change indicates running dissolution and precipitation reactions. The development of enthalpy change during heating with different heating rates is displayed in Figure 13a using the example of EN AW-6005A for T4. Therein the enthalpy levels at temperatures above the solvus temperature are defined as zero. The curves start at approximately –6 J/g (grey line) and run to 0 J/g (dotted line) due to the complete dissolution of all phases. The flatter course of the curves with increasing heating rates shows that more and more precipitation reactions are suppressed. Nevertheless, the total enthalpy change is identical.

The value of the total enthalpy change can be helpful for assessing the initial condition. If heating is performed slowly enough, the initial microstructure will dissolve completely. The characteristic enthalpy change in such cases can be defined as the enthalpy level of the initial condition. The value is expected to be constant as long as all phases are dissolved completely. In this study this holds for the T4, T6, and OA initial conditions. For these conditions, precipitates with a relatively low stability form, such as clusters, GP zones, β′′ and β′. In contrast, soft annealing leads to relatively stable precipitates—predominantly coarse (up to some 10 µm) equilibrium β (Mg_2_Si) plates. Those coarse precipitates cannot dissolve in short heating times. Therefore, in the investigated heating rate range of 0.01 to 5 K/s the dissolution of these coarse secondary particles is not finished even at 600 °C (Figure 13e).

The slower the heating the more will be dissolved and also the higher the values of enthalpy change will be. However, the enthalpy level of the SA initial state remains unknown. It is definitely larger than 15 J/g, as seen in Figure 13e.

Figure 13b–d displays the average enthalpy change of each heating rate indicated by a scatter band. Each heating rate has been repeated two to six times. Therefore, the error bars show the minimum and maximum values of all sample measurements from one heating rate. In addition, the average of the enthalpy changes of all investigated heating rates for any initial condition (T4, T6, and OA) is plotted as a straight line and an extensive scatter band indicating the standard deviation.

Table 3 specifies the enthalpy change of all the investigated aluminium alloys for the T4, T6, and OA initial states: the higher the content of the alloying elements of Mg and Si, the higher the enthalpy changes. Further, the enthalpy changes increase in the order T4, T6, OA due to the fact that more stable precipitates must be dissolved.

In this context, it should also again be pointed out that the elimination of the curvature based on the heat flow curve (see Figure 3b,e) has a significant effect on the enthalpy change. Hence, it is extremely important to proceed very careful with the elimination of bending.

**Table 3:** Enthalpy change for the specific initial heat treatment states of the investigated aluminium alloys in J/g.

Aluminium Alloy	Enthalpy change in J/g		
	Initial condition		
	T4	T6	OA
EN AW-6005A	6 ± 2	10 ± 1	12 ± 1
EN AW-6082	7 ± 3	10 ± 1	12 ± 1
EN AW-6016	3 ± 1	7 ± 1	-
EN AW-6181	7 ± 1	13 ± 1	-

## 4. Summary

The dissolution and precipitation behaviour during heating was investigated for four Al–Mg–Si alloys by means of the DSC technique in a wide range of heating rates between 0.01 to 5 K/s. In general, the analysed aluminium alloys had a similar precipitation sequence during heating. The examination of heating curves is complicated, as endothermic and exothermic reactions occur simultaneously and influence each other. The evaluation of the DSC curves for several heating rates exhibits suppressed reactions as well as a shift to higher temperatures as heating rate increases. Several initial conditions were compared and their corresponding enthalpy levels were determined. The results show a strong dependence on the initial condition. Continuous heating dissolution diagrams were created by displaying the times and temperatures of both exothermic and endothermic reactions.

## References

[1] Merklein M., Johannes M., Lechner M., Kuppert A. (2014). A review on tailored blanks—Production, applications and evaluation. J. Mater. Process. Technol..

[2] Osten J., Söllig P., Reich M., Kalich J., Füssel U., Keßler O. (2014). Softening of high-strength steel for laser assisted clinching. Adv. Mater. Res..

[3] Dutta I., Allen S.M. (1991). A calorimetric study of precipitation in commercial aluminium alloy 6061. J. Mater. Sci. Lett..

[4] Takeda M., Ohkubo F., Shirai T., Fukui K. (1996). Precipitation behaviour of Al–Mg–Si ternary alloys. Mater. Sci. Forum.

[5] Edwards G.A., Stiller K., Dunlop G.L., Couper M.J. (1998). The precipitation sequence in Al–Mg–Si alloys. Acta Materialia.

[6] Gupta A.K., Lloyd D.J., Court S.A. (2001). Precipitation hardening in Al–Mg–Si alloys with and without excess Si. Mater. Sci. Eng. A.

[7] Bryant J.D. (1999). The effects of preaging treatments on aging kinetics and mechanical properties in AA6111 aluminum autobody sheet. Metall. Mater. Trans. A.

[8] Matsuda K., Ikeno S., Terayama K., Matsui H., Sato T., Uetani Y. (2005). Comparison of precipitates between excess Si-type and balanced-type Al–Mg–Si alloys during continuous heating. Metall. Mater. Trans. A.

[9] Afify N., Gaber A., Mostafa M.S., Abbady G. (2008). Influence of Si concentration on the precipitation in Al-1 at.% Mg alloy. J. Alloys Compd..

[10] Ohmori Y., Doan L.C., Nakai K. (2002). Ageing processes in Al–Mg–Si alloys during continuous heating. Mater. Trans..

[11] Gaber A., Ali A.M., Matsuda K., Kawabata T., Yamazaki T., Ikeno S. (2007). Study of the developed precipitates in Al–0.63Mg–0.37Si–0.5Cu (wt.%) alloy by using DSC and TEM techniques. J. Alloys Compd..

[12] Chen S.P., Mussert K.M., van der Zwaag S. (1998). Precipitation kinetics in Al6061 and in an Al6061-alumina particle composite. J. Mater. Sci..

[13] Ghosh K.S., Das K., Chatterjee U.K. (2007). Kinetics of Solid-State Reactions in Al–Li–Cu–Mg–Zr Alloys from Calorimetric Studies. Metall. Mater. Trans. A.

[14] Starink M.J. (2003). The determination of activation energy from linear heating rate experiments: A comparison of the accuracy of isoconversion methods. Thermochim. Acta.

[15] Starink M.J. (2004). Analysis of aluminium based alloys by calorimetry: quantitative analysis of reactions and reaction kinetics. Int. Mater. Rev..

[16] Höhne G.W.H., Glöggler E. (1989). Some peculiarities of the DSC-2/-7 (Perkin-Elmer) and their influence on accuracy and precision of the measurements. Thermochim. Acta.

[17] Milkereit B., Kessler O., Schick C. (2009). Recording of continuous cooling precipitation diagrams of aluminium alloys. Thermochim. Acta.

[18] Höhne G., Hemminger W., Flammersheim H.-J. (2003). Differential Scanning Calorimetry: An Introduction for Practitioners.

[19] Doan L.C., Ohmori Y., Nakai K. (2000). Precipitation and dissolution reactions in a 6061 aluminum alloy. Mater. Trans..

[20] Ohmori Y., Doan L.C., Matsuura Y., Kobayashi S., Nakai K. (2001). Morphology and crystallography of β-Mg_2_Si precipitation in Al–Mg–Si alloys. Mater. Trans..

[21] Birol Y. (2006). DSC Analysis of the precipitation reactions in the alloy AA6082. J. Therm. Anal. Calorim..

[22] Birol Y. (2008). DSC analysis of the precipitation reaction in AA6005 alloy. J. Therm. Anal. Calorim..

[23] Kim S.N., Kim J.H., Tezuka H., Kobayashi E., Sato T. (2013). Formation behaviour of nanoclusters in Al–Mg–Si alloys with different Mg and Si concentration. Mater. Trans..

[24] Murayama M., Hono K. (1999). Pre-precipitate clusters and precipitation processes in Al–Mg–Si alloys. Acta Mater..

[25] Polmear I.J. (2005). Light Alloys: From Traditional Alloys to Nanocrystals.

[26] Marioara C.D., Andersen S.J., Jansen J., Zandbergen H.W. (2003). The influence of temperature and storage time at RT on nucleation of the β″ phase in a 6082 Al–Mg–Si alloy. Acta Materialia.

[27] Tsao C.-S., Chen C.-Y., Jeng U.-S., Kuo T.-Y. (2006). Precipitation kinetics and transformation of metastable phases in Al–Mg–Si alloys. Acta Materialia.

[28] Pogatscher S., Antrekowitsch H., Uggowitzer P.J. (2013). Influence of starting temperature on differential scanning calorimetry measurements of an Al–Mg–Si alloy. Mater. Lett..

[29] Starink M.J. (2007). Activation energy determination for linear heating experiments: Deviations due to neglecting the low temperature end of the temperature integral. J. Mater. Sci..

[30] Lang P., Povoden-Karadeniz E., Falahati A., Kozeschnik E. (2014). Simulation of the effect of composition on the precipitation in 6xxx Al alloys during continuous-heating DSC. J. Alloys Compd..

[31] Falahati A., Povoden-Karadeniz E., Lang P., Warczok P., Kozeschnik E. (2010). Thermo-kinetic computer simulation of differential scanning calorimetry curves of AlMgSi alloys. Int. J. Mater. Res..

[32] Falahati A., Wu J., Lang P., Ahmadi M.R., Povoden-Karadeniz E., Kozeschnik E. (2014). Assessment of parameters for precipitation simulation of heat treatable aluminum alloys using differential scanning calorimetry. Trans. Nonferr. Met. Soc..

[33] Hersent E., Driver J.H., Piot D. (2010). Modelling differential scanning calorimetry curves of precipitation in Al–Cu–Mg. Scripta Materialia.

[34] Khan I.N., Starink M.J. (2008). Microstructure and strength modelling of Al-Cu-Mg alloys during non-isothermal treatments Part 1—Controlled heating and cooling. Mater. Sci. Technol..

[35] Milkereit B., Wanderka N., Schick C., Kessler O. (2012). Continuous cooling precipitation diagrams of Al–Mg–Si alloys. Mater. Sci. Eng. A.

